# Natural Polymeric Scaffolds in Bone Regeneration

**DOI:** 10.3389/fbioe.2020.00474

**Published:** 2020-05-21

**Authors:** Miriam Filippi, Gordian Born, Mansoor Chaaban, Arnaud Scherberich

**Affiliations:** ^1^Department of Biomedical Engineering, University of Basel, Basel, Switzerland; ^2^Department of Biomedicine, University Hospital Basel, University of Basel, Basel, Switzerland

**Keywords:** natural polymer, scaffold, bone tissue, regeneration, tissue engineering

## Abstract

Despite considerable advances in microsurgical techniques over the past decades, bone tissue remains a challenging arena to obtain a satisfying functional and structural restoration after damage. Through the production of substituting materials mimicking the physical and biological properties of the healthy tissue, tissue engineering strategies address an urgent clinical need for therapeutic alternatives to bone autografts. By virtue of their structural versatility, polymers have a predominant role in generating the biodegradable matrices that hold the cells *in situ* to sustain the growth of new tissue until integration into the transplantation area (i.e., scaffolds). As compared to synthetic ones, polymers of natural origin generally present superior biocompatibility and bioactivity. Their assembly and further engineering give rise to a wide plethora of advanced supporting materials, accounting for systems based on hydrogels or scaffolds with either fibrous or porous architecture. The present review offers an overview of the various types of natural polymers currently adopted in bone tissue engineering, describing their manufacturing techniques and procedures of functionalization with active biomolecules, and listing the advantages and disadvantages in their respective use in order to critically compare their actual applicability potential. Their combination to other classes of materials (such as micro and nanomaterials) and other innovative strategies to reproduce physiological bone microenvironments in a more faithful way are also illustrated. The regeneration outcomes achieved *in vitro* and *in vivo* when the scaffolds are enriched with different cell types, as well as the preliminary clinical applications are presented, before the prospects in this research field are finally discussed. The collection of studies herein considered confirms that advances in natural polymer research will be determinant in designing translatable materials for efficient tissue regeneration with forthcoming impact expected in the treatment of bone defects.

## Introduction

Bone is an essential and multifunctional organ. While providing weight-bearing sustainment and assisting locomotion, the 206 bones in the adult human body have other defined biological roles, such as: generation of blood cells (haematopoiesis), physical protection of vital organs like brain or heart, and storage of minerals and growth factors (Clarke, 2008). Bone is constantly remodeled in physiological conditions and presents a strong regenerative capacity to react to fractures. However, in case of bone tumor removal, traumas with extensive defects or infections, the bone repair process can be impaired such that the damaged area cannot fully and spontaneously regenerate. Especially, when the defect size overcomes the healing capacity, a surgical intervention is needed. Such critical size bone defects are a clinical problem affecting millions of people worldwide, such that autologous bone graft is the second most commonly transplanted tissue after blood. Beyond the implantation of metallic prostheses, bone allo- or auto-grafts are indeed the main available therapeutic solutions for large bone defects (Laurencin et al., 2006; Calori et al., 2011). On one side, thanks to the presence of growth factors, bone material, and osteogenic cells, autologous grafts have excellent osteoinductive and osteoconductive features. Accounting for ~58% of bone substitutes, autografts remain the gold standard for small defect reconstruction, but are also associated with a number of drawbacks, like infections, bleeding, limited amount of donor bone tissue, the need for a second surgery site for bone graft harvest, donor site morbidity, and chronic pain. On the other side, ~34% of the bone substitutes have allogenic origin (often derived from cadavers). Despite being available in various forms and size, and free of morbidity issues, the allografts can cause infectious disease transmission and immunological rejection. Moreover, the sterilization procedure impairs the biological and mechanical properties of the graft (Blokhuis and Arts, 2011).

Novel strategies for the treatment and regeneration of bone tissue are offered by Tissue Engineering (TE), which aims at recovering, maintaining, and improving functions of damaged organs starting from a pool of regenerative cells, capable of both self-renewal, and differentiation in other cell types. Recent breakthroughs in the biology of stem cells (SCs), especially in relation to their trophic, regenerative and immunomodulatory functions, have raised tremendous enthusiasm in the development of innovative therapeutic solutions. Besides stem and progenitor cells, the backbone of TE relies on the investigation and optimization of cell stimulation strategies, and on the design of supporting materials (Reddy and Reddy, 2018). Thus far, several kinds of biodegradable matrices (i.e., scaffolds) have been developed to hold the regenerative cells *in situ*, re-create their biological microenvironments and sustain the growth of new tissue until integration into the transplantation area. SCs sense mechanical and biochemical signals from the niche they are located in Discher et al. (2009), such that their functional cues are orchestrated by a variety of interactions and factors, including the influence by other cells, soluble factors, and extracellular matrix (ECM). A better understanding of the complex and dynamic regulation of the cell fate specification would enable to precisely manipulate the phenotype and harness SCs for effective regenerative medicine.

However, as a composite material where hydroxyapatite (HA) crystals are dispersed into a collagen fiber matrix (Venugopal et al., 2010; Gardin et al., 2012), bone is a challenging tissue to engineer especially because of its complex hierarchical structure and dense vascularization (Logeart-Avramoglou et al., 2005; Genova et al., 2016). The first generation of synthetic bone grafts was based on metals and alloys (Ficai et al., 2011). Despite being widely used for their reproducibility and availability, such bio-inert bone implants constituted of alumina and stainless steel possess certain disadvantages, including poor integration and stiffness mismatch into the host bone, encapsulation into fibrous tissue, release of wear debris, lack of bioactivity, and bio-resorption (Ficai et al., 2011; Guarino et al., 2012). Other materials like polymers and bioactive ceramics were thus introduced to manufacture pure scaffolds first (Ficai et al., 2011; Guarino et al., 2012) and then composite ones (Sahoo et al., 2013). In composite ceramics-polymer scaffolds, the flexibility and resorption of the polymeric texture were combined to the mechanical and osteoconductive properties of ceramics (Sahoo et al., 2013). Finally, growth factors, bone morphogenetic proteins and osteogenic cells were added to increase the biological performance of the composite scaffolds (Ficai et al., 2011). In this context, the matrix of composite scaffolds can be based on polymers of either synthetic or natural origin. Since the synthetic polymers display lower biocompatibility, bioactivity, and amount of cell adhesion sites, the current research focus has shifted to the natural ones. Because of their similarity to the elements of the native ECM in the body, natural polymers have become a predominant source for the manufacturing of matrices that faithfully mimic the biological environments. Their controlled assembly can give rise to a platform of advanced supporting materials, generating systems based on hydrogels or scaffolds with customizable fibrous and/or porous architecture (Armentano et al., 2010; Wagoner Johnson and Herschler, 2011; Bonani et al., 2018).

Consisting of proteins (like collagen, silk, fibrin gels, and soy) or polysaccharides (like alginate, chitin/chitosan, starch, and hyaluronic acid derivatives) (Sell et al., 2010), natural polymers often show limited mechanical properties, immunogenic potential, or insufficient supply (Armentano et al., 2010; Swetha et al., 2010; Wagoner Johnson and Herschler, 2011). Therefore, the search for suitable materials and innovative strategies to efficiently reproduce a bone microenvironment is constantly active (Stevens, 2008; Holzwarth and Ma, 2011), and novel formulations are developed and tested to improve their *in vivo* performance (Giannoudis et al., 2005; Yunus Basha et al., 2015).

In this review, we present an overview of the various tissue constructs based on natural polymers currently developed for Bone Tissue Engineering (BTE), describing the manufacturing techniques, procedures of functionalization with bioactive molecules and their *in vitro* and *in vivo* regenerative outcomes. Finally, innovative perspectives to more faithfully mimic physiological bone microenvironments are discussed.

## Bone Morphology

To select the most appropriate biomaterial, the knowledge of the physicochemical architecture of native bone along with pertinent biomechanical features is critical. Some physico-mechanical properties of the natural bone and main BTE biomaterials are reported in Table 1.

**Table 1:** Physico-mechanical properties of natural bone and BTE biomaterials.

	**Density (g/cm^**3**^)**	**Ash fraction (%)**	**Porosity (%)**	**Pore size (μm)**	**Elastic modulus (MPa)**	**Tensile strength (MPa)**	**Elastic strain (%)**	**Compression strength (MPa)**	**Fracture toughness (MPa m^**1/2**^)**	**References**
Human cortical bone (Parallel)	1.7–2	37.7	5–10	10–50	17–18.9 GPa	124–174	1–3	130–180	6–8	(Currey, 1998; Keaveny, 1998; Fang et al., 2006; Shimko and Nauman, 2007; Clarke, 2008; Syahrom et al., 2011; BaoLin and Ma, 2014; Oftadeh et al., 2015; Michael et al., 2016)
Human cortical bone (Normal)					11.5 GPa	49	1–3			(Hamed et al., 2012)
Human trabecular bone	0.2–0.5	33.9	30–90	300–600	50–100	8	1–2	4–12		
Collagen	0.1 (skin)				46.5–35.2 (skin)					(Valero et al., 2014; Manssor et al., 2016)
	0.13 (trabecular bone)									(Ding, 2000)
					44–96			0.2–0.5		(Tanaka and van Eijden, 2003)
						~70				(Zhou et al., 2015)
Collagen fibrils					100–360 (rat tail)					(Dutov et al., 2016)
					100–400					(Varma et al., 2016)
					800					(Bhattarai et al., 2018)
Collagen triple helix					1–5.4 GPa					(Hamed et al., 2010)
					1.86 GPa. (human bone)					(Pidaparti et al., 1996)
					1.3–7.8 GPa					(Varma et al., 2016)
HA				100–600	~100 GPa	~40		>400	~1	(Hench, 1998); (Marquis et al., 2009; Chen et al., 2012)
Porous HA	0.3–1.3	100	50–90	0.8–1.4				0.2–0.4		
45 5S Bioglass®					35 GPa	42		500	0.5–1	(Marquis et al., 2009; Chen et al., 2012)

### Matrix Composition

Bone is a highly specialized connective tissue with a complex and precisely organized structure (Lafon et al., 2008; Ren et al., 2015; Arakawa et al., 2017; Lopes et al., 2018; Unal et al., 2018). From a chemical point of view, bone matrix contains around 65 wt. % mineral materials, 25 wt. % organic materials, and 10 wt. % water. The main inorganic mineral phase is represented by HA (Ca_10_(PO_4_)_6_(OH)_2_). Apart from free ions (such as ${\text{CO}}_{3}^{2-}$, Na^+^, Mg^2+^) and water, the remaining fraction is composed of organic components, type I collagen being the most abundant (nearly 90%) and serving as anchoring support for HA crystals (Lafon et al., 2008; Ren et al., 2015; Arakawa et al., 2017; Lopes et al., 2018; Unal et al., 2018). On one hand, with a length of around 50–70 nm and width of 2–5 nm, the HA crystals provide compression resistance. On the other hand, the collagen shaped in 50–500 nm diameter fibers provides bending resistance and strength during tension application (Venugopal et al., 2010; Gardin et al., 2012).

### Bone Cells

**Figure 1:**
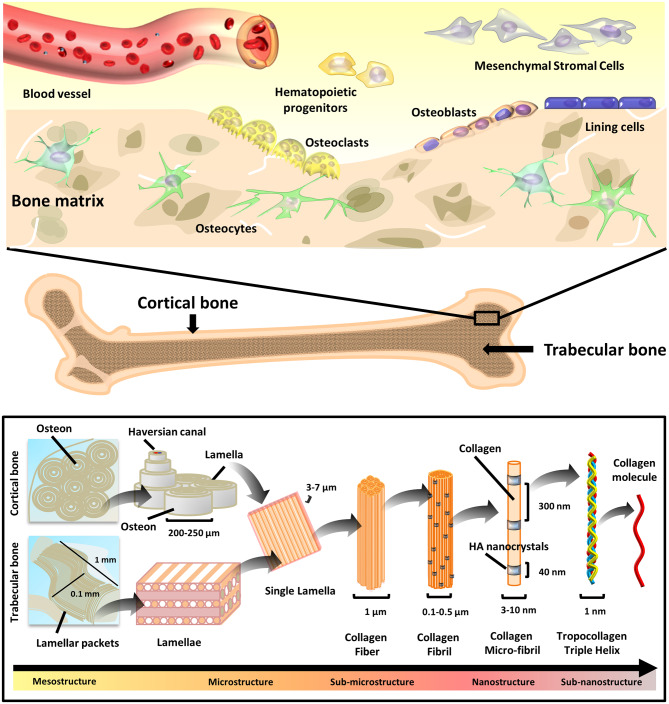
Bone tissue composition and hierarchical morphology. Bone tissue composition: cell types present in the bone tissue **(top)**, and hierarchical structure **(bottom)** of the cortical (compact) and trabecular (cancellous) bone. The various structural elements are represented, ranging from the mesostructures (i.e., osteons/lamellar packets) to the sub-nanostructures (i.e., collagen molecule).

Bone is constantly remodeled by cells with very specific functions, namely osteocytes, osteoclasts, osteoblasts, and bone lining cells (Figure 1). Bone remodeling occurs throughout life ensuring equilibrium between bone resorption and new bone formation (Hadjidakis and Androulakis, 2006). In addition to correcting micro-damages, preventing the accumulation of old, more fragile bone and maintaining plasmatic calcium homeostasis, bone remodeling also adjusts its architecture to meet new mechanical needs and adapt to external stress. Differentiating from hematopoietic cells of the monocytic/macrophage lineage, the osteoclasts are multinucleated, giant cells. Their maturation and functions are intensely influenced by the stimulating activity of the osteocytic cells (Hadjidakis and Androulakis, 2006; Nakamichi et al., 2018). In the first remodeling phase (i.e., resorption), the osteoclasts digest the mineral and organic components of old bone. Resorption is followed by repopulation by mesenchymal cells. Finally, the resorbed bone is replaced by new tissue deposited by osteoblasts (Hadjidakis and Androulakis, 2006). In fact, secreting organic and inorganic components of bone ECM and other important functional proteins, osteoblasts are bone-building cells. Deriving from multipotent Mesenchymal Stromal Cells (MSCs), the osteoblasts synthesize type I collagen, glycoproteins, proteoglycans, γ-carboxylated proteins, bone sialoprotein (BSP), osteonectin, osteopontin (OPN), and osteocalcin (OCN). Beside several growth factors, including transforming growth factor β (TGF-β), insulin like growth factor I and II (IGF-I and IGF-II), osteoblasts also produce bone morphogenic proteins (BMPs), and alkaline phosphatase (ALP) (Mackie, 2003). The newly formed organic bone matrix, not yet calcified, is called an osteoid.

Being the most abundant cell type in bone, osteocytes are terminally differentiated osteoblasts with multifunctional roles, including: (i) orchestration of osteoblast and osteoclast activity, (ii) endocrine regulation of phosphate homeostasis, (iii) sensing of local mechanical strains, and (iv) cell signaling. Residing inside lacunae within the mineralized matrix, osteocytes connect to the vasculature and bone surface by sending dendritic projections through tiny tunnels called canaliculi (Tresguerres et al., 2020).

Finally, osteoblasts can also become quiescent cells laying on the bone surface, known as bone lining cells, which are responsible for directing the mineral uptake and release at the interface with other tissues (Streicher et al., 2017).

### Architecture

Bone structure possesses a hierarchical organization (Figure 1). The overall pattern relies on the repetition of precise structural components at a nanoscale level, which then assemble into ordered micro-elements. The arrangement of micro-elements finally defines the bone macro-structure (Rho et al., 1998).

From a nanoscale point of view, bone is prominently composed of collagen fibers infiltered and surrounded by minerals. At this level, crystals and collagen fibrils are in the range of ten nanometers (nm), which are made of sub-nanostructured crystals, collagens, and non-collagenous organic proteins. Sub-nano elements account for: HA plates in the size of 2 × 25 × 50 nm, 2–3 nm thick carbonate apatite and collagen molecules with diameter of 3–10 nm (Jasiuk, 2004). The most abundant collagen is the type I, whereas the predominant crystals are of calcium phosphate (mostly HA and β-Tricalcium Phosphate, β-TCP). The mineral deposition is regulated by non-collagenous organic proteins (namely OPN, BSP, osteonectin, and OCN) that, besides functioning as chelation-regulated reservoirs of calcium and phosphorous ions, also determine the orientation and size of mineral crystals.

Scaling up from the nano to the micro-structural frame, larger structures are present. The collagen fibrils assemble into larger fibers. Lamellae, namely planar sheets resulting from the association of mineralized collagen fibers, are 3–7 μm wide. Concentric layers of few (3–8) lamellae wrapped around a central canal produce cylindrical tubes with an overall diameter of about 200–250 μm, termed as osteons or Haversian systems, running roughly parallel to the long axis of bone (Jasiuk, 2004). Size of the bone cells is also in the micro-domain, going from 1 to 2 μm for bone lining cells to 50–100 μm for mature osteoclasts (Manolagas, 2000).

Finally, the arrangement of these microstructures determines the histological nature of the bone macro-structure: the arrangement of lamellae into sinuous and irregular convolutions creates cancellous bone, namely a spongy tissue with high porosity degree, where trabeculae with typical millimeter dimensions (0.1 mm diameter and 1 mm length) are found. The regular microstructure arising from ordered cylindrical lamellae constructs impart high density to the tissue, generating the compact, cortical bone (Rho et al., 1998).

## Design of Scaffolds for Bone Tissue Engineering

**Figure 2:**
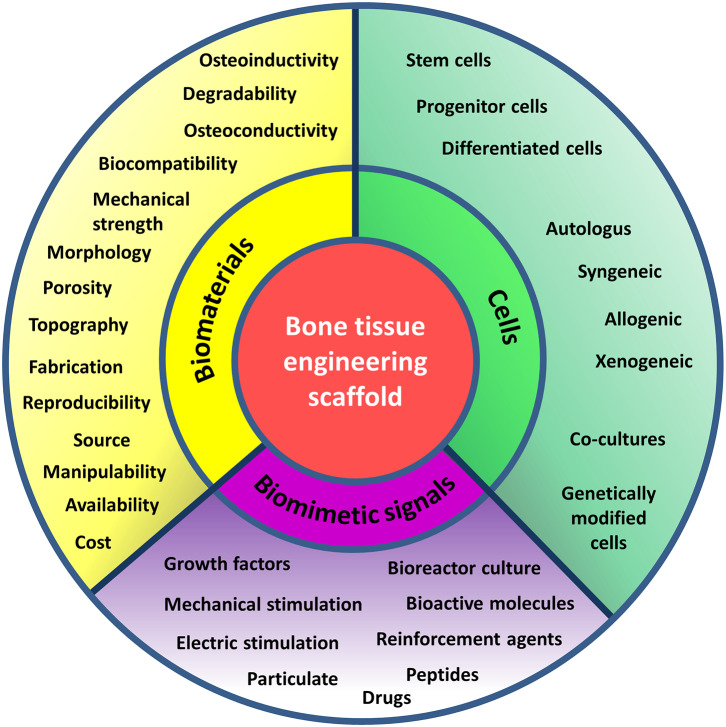
Principles of bone tissue engineering. Principles of bone tissue engineering useful in the design of scaffolds serving as functional bone substitutes. TE combines cells, signaling molecules and biocompatible materials to design bioactive scaffolds and achieve successful reinstate of a variety of tissues. The criteria to be considered in the choice of the basal material to manufacture the matrix (yellow), the cell type (green), and the inductive signals (purple) to promote bone healing and formation are recalled by keywords in the respective sections of the diagram.

Scaffolds are implemented in a damaged tissue niche and BTE principles are applied in order to trigger specific biological responses from the local environment, to induce the healing process (Figure 2). These principles consist in numerous strategies including gene therapy, drug and growth factor delivery, stem cell transplantation, and engineering of acellular scaffolds (Porter et al., 2009). The functions of BTE scaffolds consist in: (i) providing temporary mechanical support to the affected area and filling the void of bone defects, (ii) promoting the adherence and growth of circulating precursor cells and allowing for ECM deposition onto the scaffold surface (osteoconduction), (iii) eliciting vessels and bone in-growth into the porous scaffold, (iv) enhancing osteogenic differentiation via molecular signaling and new bone tissue formation (osteoinduction), (v) stimulating cell activity supporting the integration with native tissue (osteointegration), and (vi) delivering drugs or bioactive molecules to accelerate the healing process (Daculsi et al., 2013). To fulfill these functions, the scaffolds should ideally meet a number of important criteria. Besides being biocompatible, bioactive, osteoconductive, and osteoinductive, they should also maintain mechanical integrity throughout the healing process, whereas their degradation rate should guarantee the necessary mechanical support until the regeneration process is completed. Moreover, optimal cell penetration and ingrowth of vascularization into the implanted graft can occur if the structural interconnection of the internal architecture presents a pore diameter of at least 100 μm (Wagoner Johnson and Herschler, 2011). The size and shape of pores should allow for the movements of cells and the diffusion of growth factors, nutrients, along with easy excretion of by-products from the cells. Interestingly, noticing that regenerative cells specifically react to the mechanical properties of the ECM (Discher et al., 2009), it has been demonstrated that the topography and rigidity of three-dimensional (3D) microenvironments can induce adult SCs, such as MSCs, to commit toward an osteogenic lineage (Discher et al., 2009). Being dependent on traction forces, the arrangement of the adhesion ligands and organization of integrin binding can be modulated by matrix stiffness, in turn triggering the osteogenic commitment (Huebsch et al., 2010). Accordingly, tuning the physical properties of adhesion substrates has become a trend in manufacturing transplantable grafts with optimized biological features and enhanced regenerative potential (Moosazadeh Moghaddam et al., 2019). However, the nature and extent of biophysical phenomena underlying the ECM-mediated cellular fate modulation still require a deeper comprehension. Furthermore, the scaffold surface chemistry should allow for functionalization with substances fostering the new osteoid matrix formation: therefore, the specific chemical groups for conjugation with bioactive factors should be numerous and accessible, permitting the application of simple coupling procedures based on either covalent or non-covalent interactions. The material features should also allow to implement strategies for smart, controlled and sustained release of the biomolecules (Liu and Ma, 2004; Wang et al., 2018; Cui et al., 2019). Hence, the selection of the polymer is a critical aspect for scaffold design.

### Polymer Scaffolds for BTE

**Figure 3:**
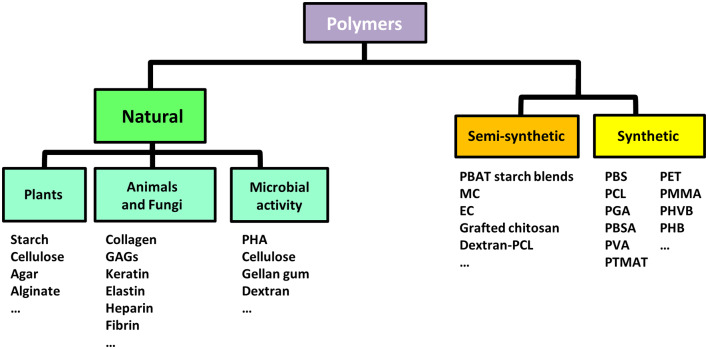
Biodegradable polymers for biomedical applications. Classification of biodegradable polymers commonly used in biomedical applications based on the origin of their source (natural, synthetic, or semi-synthetic). GAGs, Glycosaminoglycans; PHA, Poly(hydroxyalkanoates); PBAT, Poly(butylene adipate-co-terephthalate); MC, Methyl-cellulose; EC, Ethyl-cellulose; PBS, Poly(butylene succinate); PLA, Poly(lactic acid); PVA, Poly(vinyl alcohol); PGA, Poly(glycolic acid); PBSA, Poly(butylene succinate-co-adipate); PHK, Poly(hydroxylketones); PCL, Poly-ε-caprolactone; PMMA, Poly(methyl methacrylate); PHB, Poly(hydroxybutyrates); PET, Poly(ethylene terephthalate); PHVB, Poly(hydroxybutyrate-co-hydroxyvalerate); PTMAT, Poly(methylene adipate/terephthalate).

**Figure 4:**
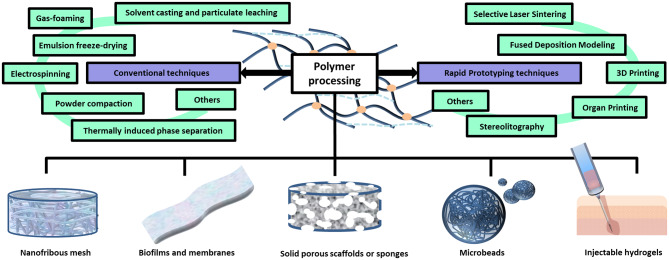
Polymer processing techniques and different scaffold architectures. Polymers can be processed through conventional or advanced techniques in order to obtain scaffolds endowed with different architectures, including: hydrogels, porous sponges, fibrous scaffolds, micro/nanoparticles and membranes.

Polymers involved in TE are either of synthetic or natural origin (Figure 3) and diverse scaffold structures can be obtained from their processing (Liu and Ma, 2004; Bhattarai et al., 2018; Rao et al., 2018; Gentile et al., 2011) (Figure 4).

On one hand, synthetic polymers have been investigated for a variety of biomedical applications, especially due to the possibility to precisely tailor their properties during manufacturing (Nemati et al., 2019). Poly(α-ester)s, polyurethanes, polyacetals, poly(ester amide)s, polyanhydrides, polyphosphazenes, and pseudo poly(amino acids), are the most eminent classes of synthetic polymers with utility in TE: for example, nanofibers of polyhydroxylketones (PHK), poly-ε-caprolactone (PCL), poly(methyl methacrylate) (PMMA), polyhydroxyl acids (PLA, PGA, PLGA), polyhydroxybutyrates (PHB), polyvinyl alcohol (PVA), polyethylene terephthalate (PET), and polyhydroxybutyrate-co-hydroxyvalerate (PHVB) can form porous, interwoven, rigid scaffolds supporting enhanced cell growth as compared to their flat films counterparts (Li et al., 2014). On the other hand, as products of the metabolic activity or other physiological processes of living organisms, the natural polymers display high biocompatibility and similarity to the natural ECM including numerous cell recognition and adhesion sites (Bonani et al., 2018; Rao et al., 2018). However, the specific source and extraction process can deeply affect their characteristics and the resulting biological activity. Because of their variability, the standardization of manufacturing procedures is critical for successful use of natural polymers in TE (Bhattarai et al., 2018; Bonani et al., 2018). Even though natural polymers can be considered as the first biodegradable biomaterials employed clinically (Nair and Laurencin, 2007), various commercially available synthetic polymers are endowed with mechanical and physicochemical features comparable to those of biological tissues (Gunatillake et al., 2006). Advantages and disadvantages associated with the biomedical use of natural or synthetic polymers are listed in Table 2 (BaoLin and Ma, 2014; Mohamed and Shamaz, 2015; Bhatia, 2016; Salehi-Nik et al., 2017).

**Table 2:** Advantages and disadvantages of natural and synthetic polymers used in scaffold fabrication for tissue engineering.

	**ADVANTAGES**	**DISADVANTAGES**	**References**
Synthetic polymers	- Defined purity and reproducible chemical/mechanical properties - Appropriate mechanical properties - Low immune response - Low production costs - Off-the-shelf availability and production in large uniform quantities - Opportunity to tailor material properties during manufacturing	- Poor biocompatibility - Risk of biodegradation side effects (nanotoxicity, inflammation, etc.) - Difficult 3D printing - Questionable cell-matrix interaction - Loss of mechanical strength after degradation (biodegradable polymers) - Low ductility - Effects of long-term permanence in the body (non-degradable polymers) - Uncontrollable shrinkage effects	(Gunatillake et al., 2006; BaoLin and Ma, 2014; Bhatia, 2016)
Natural polymers	- Natural origin - Biocompatibility - Presence of cell recognition and adhesion sites - Similarity with native ECM - Biodegradability - Not require harsh chemicals for processing - Bioresorbability - Bioactivity	- Properties dependence on extraction and processing procedures - Inadequate mechanical properties - Expensive productive methods - Susceptibility to cross-contamination - Difficult processing - Low stability	(Bhatia, 2016; Salehi-Nik et al., 2017)

Porous 3D scaffolds for BTE can be fabricated by processing the polymers through different technologies (Figure 4). The traditional ones include: solvent casting and particulate leaching, gas foaming, emulsion freeze-drying, electrospinning, and thermally induced phase separation (Liu and Ma, 2004). The “solvent casting and particulate leaching” method simply requires to add certain water soluble salt particles (e.g., sodium chloride, sodium citrate) into a solution of biodegradable polymers, which is then casted into a mold of the defined shape. After removing the solvent by evaporation or lyophilisation, pores are formed by leaching out the salt particles (Ma and Langer, 1998). In gas foaming, a gas (usually carbon dioxide) is applied at elevated pressure to solid polymer disks until reaching saturation. Then, the sudden release of the gas causes thermodynamic instability of the polymer system, enabling several gas bubbles to nucleate and grow inside the material, which eventually define a spongy structure (Harris et al., 1998). In emulsion freeze-drying, a polymer solution in organic solvent and water are homogenized and rapidly cooled down to preserve the liquid state structure. Solvent and water are eliminated by freeze-drying, leaving a structure with high porosity degree (even greater than 90%) (Whang et al., 1995). Electrospinning is a technique where high electrostatic forces are used to squeeze a viscoelastic solution into jet, overcoming its internal cohesive forces: upon solvent evaporation, nano/micro sized fibers are formed (Bürck et al., 2018). In sol-gel technique, inorganic metal salts or metal organic compounds are dissolved in a solvent in order to allow a colloidal suspension (namely a sol) to form as a consequence of a series of hydrolysis and polymerization reactions. After being casted in a mold, the sol turns into a wet gel which is then subjected to heat treatment to produce dense glass or ceramic articles (Xing et al., 2010). Initially used for the preparation of porous membranes and then 3D scaffolds, the controlled thermally induced phase separation technique accounts for a first processing step where the polymers are dissolved into solvent at high temperature. Afterwards, lowering the temperature induces a solid-liquid or liquid-liquid phase separation. Finally, the solidified solvent-rich phase is removed via sublimation, leaving hollow spaces determining the matrix porosity (Zhang and Ma, 2002). Other traditional fabrication techniques include: fiber mesh, fiber bonding, melt molding, and powder compaction methods (Garg et al., 2012). More advanced procedures are based on a rapid prototyping approach (also known as *solid free form* fabrication), such as in 3D printing, where specific objects or components are produced by ink-jet printing a binder onto sequential powder layers (Yang et al., 2002). Other systems that fall under the rapid prototyping category are: stereolitography, selective laser sintering, fused deposition modeling, organ printing, and membrane lamination, which have been reviewed somewhere else (Garg et al., 2012).

Depending on the final use of the scaffolds, different mechanical behaviors might be required such that the choice of polymers should be adapted also accordingly. For instance, due to the difficulty of reaching the target areas, injectable and soft materials are preferred in dentistry, where stents have to fit the small deposition sites in the pulpo-dentinal complex and periodontal apparatus (Chieruzzi et al., 2016). In this context, polymers with gelation ability are suitable. The surgical reconstruction of bone and cartilage instead requires larger grafts simulating the precise mechanical properties of hard tissues: this effect can be obtained through a well-balanced composition of bone-bioactive inorganic substances (like HA, bioactive glasses, and calcium phosphate) and polymers. Especially, the natural ones (such as collagen, chitosan, and silk fibroin) are preferred to generate soft matrices with great potential in BTE, thanks to a high biodegradability, cytocompatibility and a minimal immunogenicity (Bhattarai et al., 2018; Rao et al., 2018). Furthermore, besides being the main structural component in scaffolds, another relevant function of BTE polymers consists in delivering bioactive molecules and drugs (Zamani et al., 2010). In biodegradable polymers, the kinetics of degradation occurring under physiological conditions depends on the nature of polymer blends (copolymer or pristine), architectural scale (micro or nano-scale), and presence of reaction accelerators or inhibitors (La Mantia et al., 2017). Beyond that, various mechanisms for controlled release of biofactors can be implemented: for instance, the instability of the drug-matrix linkage can derive from hydrolysis or enzymatic digestion, resulting into tunable biodegradation rate (Nair and Laurencin, 2007).

### Natural Polymers in BTE

Natural polymers can be either protein or polysaccharide-based (Garg et al., 2012). Unlike polysaccharides, proteins present the amino acid sequences typically associated with cell attachment via integrin-binding domains. Therefore, cell adhesion and osteoconductivity have to be enhanced in polysaccharidic scaffolds by chemical surface modifications (Luna et al., 2011), mixing with osteoconductive materials, and incorporation of integrin-binding sequences or cell adhesion proteins (Kowalczewski and Saul, 2018). The most commonly studied polymers of natural origin for BTE are collagen/gelatin, alginate, chitosan, silk, hyaluronic acid, elastin, glycosaminoglycans (GAGs), peptides, and others (Vagaská et al., 2010; Jahan and Tabrizian, 2016). Concentration, conditions of polymerization and the introduction of functional groups allow for a modulation of the porosity, charge, and mechanical strength of natural polymers, as well as the addition of chemicals, proteins, peptides, and cells enables to control their bioactivity (Lee and Shin, 2007; Lee and Yuk, 2007). In the following paragraphs, the properties and use of polymers will be discussed, highlighting recent studies where these materials have been modified in different manners in order to improve their osteogenic capabilities. Although various polymeric materials have been already investigated, no single biodegradable polymer can meet all the requirements for application in BTE: the advantages and disadvantages of the most widely used polymers are summarized in Table 3.

**Table 3:** Advantages and disadvantages of various natural polymers used in fabrication of BTE scaffolds.

	**ADVANTAGES**	**DISADVANTAGES**	**References**
**Protein-based polymers**			
Collagen	- Biocompatible - Biodegradable - Fibrous morphology - Non-toxic - Non-antigenic - Mimic native bone ECM topography - Biologically renewable - Bioadhesive - Biofunctional - Ability to be cross-linked	- Poor mechanical properties - Low stability - Fusion of nanofibers in aqueous environment - Low melting point - Viral and prion contamination - Difficult processing - Difficult control over extent and rate of degradability - Potentially damaged by sterilization methods - Expensive if produced by recombinant technologies	(Whang et al., 1999; Dong and Lv, 2016; Zhang et al., 2017)
Gelatin	- Biocompatible - Biodegradable - Anti-thrombogenic - Good cell recognition properties - Low antigenicity - Easy to mold into a range of shapes (injectable hydrogels and sponges)	- Low stability - Chemical cross-linking needed - Poor mechanical properties - Brittleness	(Garg et al., 2012; Echave et al., 2017)
Silk fibroin	- Biocompatible - Biodegradable - Slow degradation - Excellent mechanical properties - High thermal stability - High mechanical strength	- Reduced availability (*e.g*. low production from spiders) - High brittleness - Residue contaminants	(Shi et al., 2016; Kowalczewski and Saul, 2018)
Hyaluronic acid	- Highly biocompatible - Biodegradable - Excellent viscoelasticity - Excellent water solubility - Natural component of ECM and structurally similar to GAGs - Easy and controllable production in a large scale via microbial fermentation - Easy functionalization - Negatively charged - Easy manipulation	- Difficult processing by electrospinning (due to high viscosity and surface tension) - Poor mechanical properties - Expense of preservation and storage in a cryo-freezer.	(Khan and Ahmad, 2013; Bae et al., 2014; Shi et al., 2016)
Peptides	- Biocompatible - Biodegradable	- Poor mechanical properties	(Mata et al., 2010; Visser et al., 2016)
Keratin	- Biocompatible - Biodegradable	- Poor mechanical properties	(Tachibana et al., 2005; Sayin et al., 2014; Kowalczewski and Saul, 2018)
Fibrin	- Biocompatible - Biodegradable - Insoluble into water - Improved cellular interaction	- Low integrity and rapid degradation *in vivo*- Instability - Low mechanical stiffness	(Garg et al., 2012; Noori et al., 2017)
Chondroitin sulfate	- Non-toxic degradation products (oligosaccharides) - Non-immunogenic	- Readily water-soluble nature	(Garg et al., 2012; Farrugia et al., 2018)
Spongin	- Biocompatible - Inexpensive - Low risk of transmission of infection-causing agents - Widely available - Well-established farming techniques - Appropriate porosity and surface chemistry - Stable *in vitro*	- Need for determining aquaculture systems or farming (when *ex situ* cultivation is difficult) - Species-dependent variability of characteristics and composition	(Green et al., 2003; Granito et al., 2016)
Heparin	- Preserve the growth factor stability and bioactivity	- Reduced cell growth rate	(Chung and Park, 2007)
**Polysaccharide-based polymers**			
Chitosan	- Biocompatible - Biodegradable - Biologically renewable - Non-toxic - Non-antigenic - Inexpensive - Positively charged - Antibacterial properties	- Difficult processing by electrospinning - Immunogenicity - Long delay in bone formation (after several months or years) - Relatively weak mechanical strength and stability	(Garg et al., 2012; Levengood and Zhang, 2014; Shi et al., 2016)
Alginate	- Biocompatible - Biodegradable - Simple gelation methods - Crossilinkable and injectable - Easy functionalization - Resistance to acidic conditions - Negatively charged - Adjustable properties based on the two monomer content	- Poor mechanical properties - Leaching of entrapped drugs - Uncontrolled degradation kinetics - Difficult sterilization and handling	(Shi et al., 2016; Kowalczewski and Saul, 2018)
Starch	- Biodegradable - Abundant - Overwhelming - Biological renewability	- Brittleness - Highly difficult processing - Destruction or reorganization of the structure of the semi-crystalline native starch granules	(Martins et al., 2009; Khan and Ahmad, 2013)
Agar	- Biocompatible - Biodegradable	- Difficult processing - Difficult extraction	(Garg et al., 2012; Witzler et al., 2019)
Dextran	- Biocompatible - Biodegradable - Several derivatives with different molecular weights are readily available	- Risks of coagulation abnormalities - Cost - Over hydration - Risk of anaphylaxis	(Garg et al., 2012; Nikpour et al., 2018)
Cellulose	- Biocompatible - Inexpensive - Readily available - Easily converted into derivatives - Porous	- Long renewal time - Low degradability *in vivo*	(Khan and Ahmad, 2013; Sofi et al., 2018)
Carrageenans	- High molecular flexibility - Thixotropic nature	- Gel dissolution in the absence of a gel-inducing reagent - Elevated melting temperature	(Garg et al., 2012; Yegappan et al., 2019)
Gellan gum	- Resistance to acidic conditions and high temperature - Transparency, flexibility and elasticity in the highly acylic gels	- Low elasticity and brittleness when used in the low acyl form	(Pereira et al., 2013; Manda et al., 2018)

#### Collagen

##### Structure and characteristics

The collagens are animal-derived fibrous glycoproteins. In vertebrates, they account for 28 distinct types coded by at least 45 different genes (Meyer, 2019). As a major constituent of the ECM in various connective tissues, collagens are the most abundant protein in the human body (25 to 35% of the whole-body protein content), and as a primary component in bone, they are also ideal candidates for 3D scaffold design (Aravamudhan et al., 2013; Dong and Lv, 2016). In tendon, bone, skin, and cartilage, a restricted number of collagen types dominate: while cartilage mainly consists of type II collagen, collagen type I is prevalent in skin, tendon and bone (beside the less frequent types III and V). The collagen molecule structurally consists of a triple helix of elongated fibrils, known as a collagen helix. The helix can contain the same or very similar polypeptide chains leading to homo- or hetero-trimers (Gross et al., 1955; Bürck et al., 2018). In natural bone, collagen fibrils serve as a template for mineralization. Biocompatible, bioactive, and rich in surface-binding sites for cells, collagen stimulates cell adhesion, proliferation, and differentiation, representing therefore an excellent substrate for scaffold fabrication.

##### Manufacturing and properties of collagen scaffolds

Collagen can be shaped into nanofibers via electrospinning (Matthews et al., 2002; Bürck et al., 2018). Different solvents, such as hexafluoroisopropanol (HFIP), mixture of formic and acetic acid, and diluted acetic acid, have been used to electrospin collagen for nanofibrous scaffold preparation (Ekaputra et al., 2011; Dippold et al., 2017; Dulnik et al., 2018). To form BTE scaffolds, collagen can also be used in the form of hydrogel. The polymerization conditions, such as pH, collagen type and concentration, affect the microstructure of the resulting matrices, acting on the fibril diameter and density (Roeder et al., 2002). Also, the hydraulic permeability of collagenous scaffolds can be manipulated to optimize not only the internal oxygen flow and nutrient exchange, but also the overall mechanical properties of the construct and the cell-scaffold interactions. Depending on pore size, number, orientation, distribution and interconnectivity, the ability of collagen hydrogels to transfer fluids through their interstices varies under applied pressure. Moreover, the collagen fibril density and the hydraulic permeability of the gel inversely correlate: it was demonstrated that in gels with higher fibril densities and low hydraulic permeability, the application of a plastic static compression greatly increases their modulus and reduces the MSC-induced gel contraction (Serpooshan et al., 2010). Moreover, even though the influence of hydraulic permeability on the osteoconduction and osteoinduction remains to be fully defined, evidences of improved MSC proliferation, differentiation, and mineralization were found in compressed collagens, appearing therefore promising for bone grafting purposes. In another study, scaffolds composed of type 1 and 3 collagen hydrogels in the ratio 9:1 were seeded with MSCs from different sources to assess their ability to undergo osteogenic differentiation (Schneider et al., 2010). On this model, the cells were able to migrate, colonize and remodel the matrix through the secretion of matrix metalloproteinases (MMP), inducing its strengthening and contraction. As compared to other polymers (like alginate), collagen hydrogels showed increased binding ability for co-cultures of MSCs and endothelial cells (ECs) useful in studying pre-vascularization of engineered bone (Nguyen et al., 2017). These systems, in which an amplified gene and protein expression of osteogenic and angiogenic markers was demonstrated, further increased by bioreactor-induced shear stress, are expected to advance the design of microenvironments for bone tissue substitutes.

Nevertheless, pure collagen scaffolds are fragile, such that direct cell culture causes extensive gel contraction and unstable geometrical properties (Mizuno et al., 2000; Otsuka et al., 2013). Additionally, they owe insufficient bioactivity to foster the cell bone forming ability and poor mechanical strength to sustain bone regeneration, often making it necessary to add polymers and other biomolecules in order to improve osteoinductivity (Harley et al., 2007). For instance, the low Young modulus value of collagen (see Table 1) can be increased by crosslinking with synthetic polymers (Bhattarai et al., 2018). In another study, to compensate poor mechanical properties, a collagen hydrogel was inserted onto a macro-channeled PCL scaffold fabricated via robotic dispensing technique (Yu et al., 2012). The growth and osteogenesis by MSCs were studied in a perfusion bioreactor, demonstrating upregulation of genes involved in mechanotransduction, the process by which physical forces are converted into biochemical signals that are essential for bone formation (Rosa et al., 2015).

##### Biocomposites

A variety of bioactive compounds, including bioceramics, carbon, and polymer materials, have been incorporated into collagen to prepare composite scaffolds in which porosity, structural stability, osteoinductivity, and osteogenicity are improved (Zhang et al., 2017). Calcium phosphate (CaP) and calcium silicate (CaSi) bioceramics are combined with collagen to simulate the intrinsic inorganic compartment of bone. Based on direct mixing and co-precipitation principles respectively, the suspension and immersion techniques are the main manufacturing methods to integrate bioceramics into collagen (Kikuchi et al., 2004; Yunoki et al., 2007; Xia et al., 2016). Some examples of collagen biocomposites and related properties are reported in Table 4.

**Table 4:** Physical properties of BTE scaffolds based on biocomposites with natural polymers.

**Polymer**	**Composite**	**Manufacturing method**	**Density (mg/cm^**3**^)**	**Porosity (%)**	**Pore size (μm)**	**Elastic modulus (MPa)**	**Tensile strength (MPa)**	**Compression modulus (MPa)**	**Compression strength (MPa)**	**References**
Collagen	Milli-HA	Compression molding		85	300–400			1		(Kane et al., 2015)
	nHA	Freeze drying	≤79.4	84.9– 96.5	50–150			≤671.7 kPa		(Sionkowska and Kozlowska, 2013)
	nHA	Immersion method		98.9				4 kPa		(Tampieri et al., 2003)
		Freeze drying		99.4				5.5 kPa		
	PLGA-nHA	Layer by layer solvent casting				1.2 GPa	9.7			(Liao et al., 2007)
	PCL-nHA	Electrospinning		85.6		1.73				(Venugopal et al., 2007)
	GAG–CoBG	Freeze drying		98				6 kPa		(Quinlan et al., 2015)
Gelatin	Milli-HA	Freeze drying		56		0.8 GPa			14	(Landi et al., 2008)
	nHA	Electrospinning				412	4.4			(Kim et al., 2005)
	nHA	Layer by layer solvent casting				8	1.8			(Hamlekhan et al., 2011)
	nHA-PCL					23.5	3.7			
	αTCP	Freeze drying			350–170			4.5	0.4	(Panzavolta et al., 2009)
Chitosan	nHA	Freeze drying				9 kPa				(Thein-Han and Misra, 2009)
	nHA	Fusing microspheres		33.7		117.57				(Chesnutt et al., 2009)
	nHA	Co-precipitation							61	(Liuyun et al., 2008)
	nHA	*In-situ* precipitation				704			23	(Cai et al., 2009)
	nHA-PLA			85		880			266	
	Gel-nHA	Freeze drying		70.8			4			(Li et al., 2011)
	Gel–Pectin–nHA			79			17.4		13.45	
Alginate	HA-CS	*In situ* co-precipitation	0.12–0.24 g/cm^3^	84.9–74.5	70–200					(Jin et al., 2012)
	CS	Freeze drying		94.5						(Venkatesan et al., 2014a)
	CS-fucoidan			94.9						
Silk fibroin	nHA	Solvent casting						1		(Bhumiratana et al., 2011)
	Wollastonite	Freeze drying		81.8				2	0.2	(Zhu et al., 2010)

It is well-established that adding HA not only increases the compression modulus of collagen scaffolds, but also provides a larger and rougher adherence surface allowing for improved adhesion, bioactivity, and proliferation of cells (Sionkowska and Kozlowska, 2013). Collagen scaffolds enriched with HA modified by chemical substitutions with Mg^2+^ can exert a regulatory effect on bone formation process (Kikuchi et al., 2001; Calabrese et al., 2016), while Zn-based substitution stimulates the expression of osteogenesis-related genes and cell proliferation (Minardi et al., 2015). After years of exploration, collagen-HA composites with controllable micropore organization and interconnectivity, isotropic equiaxed structures and tunable properties can be manufactured (Xia et al., 2013), paving the way to a systematic production of tailored substitutes fitting the bone defect shapes thanks to the development of the 3D printing technology. β-TCP has also been widely employed to prepare collagen composites for bone regeneration (Arahira and Todo, 2016). With respect to HA, β-TCP displays lower Ca/P ratio (≈1.67 and 1.5, respectively) and faster degradation rate, leading to complete replacement in newly formed bone tissues (Cao and Kuboyama, 2010). Five to 10 wt. % has been recommended as an optimal β-TCP dose to achieve appropriate mechanical stiffness and release of Ca^2+^ ions in collagen scaffolds, eventually supporting cell proliferation, neovascularization and bone formation *in vivo* (Murakami et al., 2017). Cerasorb® Ortho Foam, a type of collagen-β-TCP composite, was used to fill bone defects of critical size in a rabbit distal femoral condyle model, stimulating bone healing, and causing neither toxic nor immunological reactions (Zheng et al., 2014). In this study, the swelling of collagen was also found to support a better contact of the implant with the surrounding tissue.

Among silica-based osteoconductive and osteoinductive glass biomaterials, bioactive glasses (BGs) are structurally based on SiO_2_-CaO-P_2_O_5_ networks. Besides promoting both osteogenesis and angiogenesis *in vitro* and *in vivo* (Xynos et al., 2001; Gorustovich et al., 2010), addition of BGs generates collagen composites with high-performance in mimicking bone mineralization, given that the release of Ca, P, and Si causes Ca and P precipitation at the surface of the implants with consequent deposition of amorphous Ca-P crystals. The crystals finally transform into hydroxycarbonate apatite (HCA) by dehydration (Izquierdo-barba et al., 2008). Similarly, the wollastonite (CaSiO_3_) releases Si and Ca ions stimulating osteogenic differentiation and cell proliferation, and inducing the deposition of bone-like apatite on their surface after soaking in simulated body fluids (SBF) (Li and Chang, 2005, 2013; Wang X. et al., 2015). But even though wollastonite enhances mechanical strength of the constructs, bone regeneration ability and angiogenesis, more investigations are needed to define the bioactivity, osteogenic potential, and immunogenicity of collagen composites upon *in vivo* implantation.

To improve osteoinduction, osteointegration, structural stability, and mechanical properties, other materials can be combined to collagen. For instance, carbon nanotubes (CNTs) are reported to augment the tensile strength, the stress resistance and the apatite deposition ability in collagen composites, as well as to the enhance osteogenic differentiation of MSCs (Usui et al., 2008; Da Silva et al., 2009; Baktur et al., 2013). Collagen can also be crosslinked with flakes of graphene oxide (GO) by 1-ethyl-3-(3-dimethylaminopropyl) carbodiimide hydrochloride (EDC) (Kang et al., 2015), generating scaffolds with an elastic modulus (38.7 kPa) comparable to that of developing bones (≥30 kPa in osteoids) (Lo et al., 2000) and higher than that of unmodified collagen (14.6 kPa). The enhanced matrix stiffness over-activated FAK and ERK intracellular signaling pathways in cultured MSCs, boosting the focal adhesion formation, cytoskeletal tension, proliferation rate, and osteogenic differentiation via increased expression of ALP and OP. Among naturally derived polymers, GAGs, and silk fibroin are the most frequently used in collagen composites (Farrell et al., 2006; Wang et al., 2011; Alhag et al., 2012). Especially, GAGs generate suitable collagenous 3D environments for the osteogenic stimulation of MSCs, with promising results for bone tissue regeneration both *in vitro* and *in vivo* (Farrell et al., 2006; Tierney et al., 2009; Alhag et al., 2012).

Also serving as basic scaffold components alone (like collagen) as well as reinforcement elements in scaffolds constituted of other materials, various biodegradable synthetic polymers (such as PCL, PGA, PLA, PVA, PET, etc.) have been extensively employed to fabricate collagenous BTE materials (Fujita et al., 2005; Stratton et al., 2016; Weisgerber et al., 2016; Zhang et al., 2017). Importantly, a HA-Col-PCL composite scaffold with unique nano-micro-macro hierarchical structure was prepared by Wang J. et al. (2015). A PCL support, manufactured by means of a rapid prototyping technology, provided a macroporous framework further filled with collagen. In turn, collagen offered a microporous architecture where HA nanocrystals were deposited via biomimetic mineralization. Beyond displaying a compressive modulus similar to cancellous bone (68.75 ± 3.39 MPa) falling within the suggested value range for BTE (10–1,500 MPa) (Hollister, 2005), this composite showed remarkable osteoinductive properties and rapid bone regeneration in a rabbit radius model. To further increase their interaction with the hydrophilic collagen, the hydrophobic synthetic polymers can be chemically modified. For example, surface activation of PLA fibers with diamine and glutaraldehyde induced stronger interaction between the two phases, resulting in an increased compression modulus of collagen, in which the cells spread and attached more efficiently (Hiraoka et al., 2003).

##### Sources of collagen and commercial biocomposites for BTE

Various collagen types are found in the tissues of mammals, reptiles, fishes, etc. (Nomura et al., 1996; Wood et al., 2008; Pati et al., 2010; Addad et al., 2011; Parenteau-Bareil et al., 2011; Ferreira et al., 2012; Kim et al., 2013; Ferraro et al., 2017; Felician et al., 2018; León-López et al., 2019). In the mammalian body, the tendon and the skin, rich in fibrous collagen, are used as main sources for collagen extraction (Ferreira et al., 2012). Even though collagen sponges obtained from bovine, ovine, and porcine tendon have reported to share similar physico-mechanical properties (Ghodbane and Dunn, 2016), it has to be mentioned that, in general, the xenograft origin represents a key factor determining the cultural accommodation of biomedical products (Easterbrook and Maddern, 2008).

Some sponges are commercially available for the treatment of long bone fractures (Garg et al., 2012; Kuttappan et al., 2016). InFuse is a collagen sponge marketed by Medtronic Sofamor Danek (Memphis, TN) in the U.S., serving as carrier for BMP in clinical therapy of spinal fusion. Also an osteoconductive matrix made of crosslinked collagen type I fully coated with HA, named Healos and distributed by DePuy Orthopedics, Inc. (Warsaw, IN), is applied in spinal fusion treatment. Biomend is a collagen membrane conventionally used in the regeneration of periodontal tissue, commercialized by Integra Lifesciences Corp. (Plainsboro, NJ). Despite the risk of possible iatrogenic transmission of prion-related diseases to patients treated with bovine grafts (Kim et al., 2013), collagen type I of bovine origin has been predominately applied in BTE (Ferreira et al., 2012; Ferraro et al., 2017). In 1993, Collagraft™ (Collagen Corp., USA), namely HA and TCP-enriched bovine collagen seeded with the patient's bone marrow, received the FDA approval becoming the first collagen-based implant for bone. In parallel, other xenograft types have been investigated (Salamanca et al., 2018). Collapat® (BioMet Inc.) is a composite of HA and type I collagen from the calf skin recommended for the treatment of aseptic enclosed metaphyseal bone defects. Its application accelerates the bone regeneration rate of five times, such that a complete closure of rabbit femoral defects can be achieved in 4 weeks (Katthagen and Mittelmeier, 1984; Yunus Basha et al., 2015). Composed of porcine type I and type III collagen fibers, BioGide (GeistlichPharma AG, Wolhusen, Switzerland) is the most eminent commercial collagen membrane, showing marked regenerative abilities in several rat studies (Zhao et al., 2000; Taguchi et al., 2005). OsteoBiol® mp3, namely collagenized porcine bone xenografts, proved to be biocompatible, bioabsorbable, and osteoconductive over a 4-month period following their insertion in rabbits' tibiae (Calvo Guirado et al., 2013). Other FDA approved orthopedic products based on highly purified type I collagen from cattle include composite matrices processed into strips, blocks, and pads for bone grafting procedures (OssiMend® Bioactive Moldable Strips, purchased from Collagen Matrix, Inc. New Jersey, USA) and fibrillar collagen dental dressing (Collatene™ Fibrillar Collagen Dental Dressing, commercialized by Ace Surgical Supply Co., Inc., MA, USA) (Ferreira et al., 2012; Salamanca et al., 2016, 2018). GMP-grade constructs for the treatment of degenerative bone disease with limited involvement of skeletal tissue are obtained from Gingistat® (Vebas, Milan, Italy), a clinical grade biomimetic sponge made of lyophilized collagen of equine origin (Donzelli et al., 2007). Gingistat® has been chosen as basic material for regenerative constructs opposing to alveolar bone resorption in the periodontal disease (Salvadè et al., 2007).

##### Advanced biological functions of collagen scaffolds

Current studies investigate: (i) integrated approaches combining more than one material with the collagen structures, (ii) loading with various cells, and (iii) enrichment with biofactors. The interaction of osteoblasts, pre-osteoblasts, and SCs with collagen inside BTE constructs was characterized *in vitro* and confirmed with *in vivo* proofs of principle. In injectable form, collagen hydrogel is a useful delivery platform for bioactive molecules (mostly, chemicals and proteins, but also nucleic acids). This is due to its ability to swell without disintegrating and to incorporate hydrophobic drugs, as well as to a tuned degradation rate allowing for a controlled release. Release profiles of bFGF from collagen hydrogels were monitored and studied in relation to the proliferation ability and osteogenesis of seeded MSCs (Oh et al., 2012), leading to the identification of an optimal bFGF dosage (10 ng/ml) for the preparation of highly stimulatory constructs. In another study, the spatial immobilization of bone morphogenetic protein-4 (BMP4) into collagen-PLGA hybrid platforms improved Ca deposition and expression of osteogenic marker genes (such as type 1 collagen, OPN, and OCN) after *in vivo* ectopic implantation (Lu et al., 2012). In addition, silicified collagen scaffolds loaded with SDF-1 formed bone upon subcutaneous implantation (Niu et al., 2012). Confirming results from *in vitro* transwell migration experiments, in which both MSCs and endothelial progenitor cells (EPCs) showed improved migration with higher concentrations of released factor, cell-free SDF-1 containing hydrogels stimulated cell-homing *in vivo* and enhanced blood vessel formation.

#### Gelatin

##### Structure and characteristics

Gelatin derives from an irreversible hydrolysis of collagen, reducing 300 kDa protein fibrils into smaller peptides, whose molecular weight range varies depending on the chosen denaturation method (Young et al., 2005). The amino acid content of gelatin reflects that of collagen, namely 19 amino acids, with prevalence of glycine, proline, and hydroxyproline. Due to a good degradability, solubility, biocompatibility, and easy supply, gelatin is widely used in biomedicine (Echave et al., 2017). It presents lower antigenicity with respect to complete collagen. Moreover, collagen is rich in RGD residues, namely Arg-Gly-Asp tripeptide sequences known to promote cellular adhesion, but these only become accessible after fiber processing or degradation (Barczyk et al., 2010). Its denaturation to gelatin reveals RGD sequences favoring adhesion, proliferation, and differentiation of cells. Furthermore, gelatin also maintains a matrix metalloproteinase (MMP) recognition sequence enabling enzymatic degradation (Van Den Bulcke et al., 2000). Its chemical and physical properties can be tuned flexibly to meet the requirements of various applications. For instance, the isoelectric point can vary depending on the processing method (acidic-processing or alkaline pre-treatment processing), yielding either a basic or an acidic gelatin with positive and negative surface charges, respectively (Young et al., 2005). Crosslinking by glutaraldehyde immersion induces mechanical strengthening of the gelatin electrospun fibers, exhibiting almost a 4-fold higher value of Young's modulus as compared to integral collagen (174 vs. 52 MPa) (Matthews et al., 2002; Huang et al., 2004).

##### Manufacturing of biocomposites and their properties

Nanofibers of gelatin have been produced via electrospinning also by applying a water based co-solvent approach (Song et al., 2008). Different concentrations of HA were combined with gelatin electrospun fibers to fabricate scaffolds for investigation of the fate of human fetal osteoblasts (Salifu et al., 2017). PCL and gelatin have been co-assembled into nanofibers used in BTE applications (Naghieh et al., 2017). Biodegradable grafts for bone defect treatment based on injectable enzymatic cross-linkable gelatin combined with functionalized gold nanoparticles have also been proposed, which also could serve as a good template for drug and cell delivery for TE (Lee et al., 2018). Varying the proportion among components in the formulations of gelatin composites with chito-olisaccharides (COS) and magnesium calcium phosphate (MCP) was found to regulate the scaffolds' pore size with a direct effect on osteogenic differentiation (Ratanavaraporn et al., 2011; Hussain et al., 2012).

##### Chemical modification

Certain shortcomings, such as the insufficient thermostability and the potential poisonousness of chemical crosslinking, can be overcome by functional modification of the gelatin structure. One of the most interesting modified gelatin is obtained by reaction with methacrylic anhydride (MA) (Van Den Bulcke et al., 2000). A large number of amino groups exposed on the side chains of gelatin are thereby replaced by methacryloyl groups. MA-gelatin acquires the capacity to photo-crosslink, producing hydrogels with excellent thermostability. These have recently emerged for their applicability in BTE, either alone or in combination with other biomaterials (Heo et al., 2014; Zuo et al., 2015; Celikkin et al., 2018; Raina et al., 2019). Studies concerning the bone mineralization have been carried out with MA-gelatin hydrogels. Zhou et al. claimed a control of the mineralization by the degree of methacrylation, while Zuo et al. developed and compared two distinct methods (i.e., circle-and-cross and a layer-by-layer methods) to build osteon-like structures in bionic bones (Zuo et al., 2012; Zhou et al., 2014).

#### Chitosan

##### Structure and characteristics

Chitosan (CS) is a natural polysaccharide deriving from chitin, the major component of crustacean exoskeleton (Khor and Lim, 2003; Levengood and Zhang, 2014). It consists in a copolymer of (1 → 4)-2-acetamido-2-deoxy-β-D-glucan (N-acetyl D-glucosamine) and (1 → 4)-2-amino-2-deoxy-β-D-glucan (D-glucosamine). CS is commonly extracted from marine crustacean shells through chemical hydrolysis (which accounts for subsequent phases of demineralization, deproteinization, discoloration, and deacetylation), but it can also be isolated by enzymatic digestion of the cell walls of certain fungi (Shahidi and Abuzaytoun, 2005; Cai et al., 2006; Kim and Rajapakse, 2005). The cationic nature of CS favors the interaction with negatively charged molecules like GAGs, proteoglycans, and other nutrients. Despite a lack of mechanical strength, a fast degradation rate and the absence of cell signaling molecules, CS is a biodegradable and biocompatible material owing adhesive and antibacterial properties, particularly appealing in wound healing (Khor and Lim, 2003). Nevertheless, the combination with polymers, cells, and bone-inducing factors can impart osteogenicity (Martins et al., 2010).

##### Manufacturing and properties of CS scaffolds

Being structurally similar to GAGs, CS was found suitable to manufacture highly porous scaffolds with interconnected pores, with good bone ECM-mimicking ability and permissive to bone ingrowth into the graft (Khor and Lim, 2003; Di Martino et al., 2005; Pang et al., 2005). CS can be easily shaped into various forms like sponges, films, fibers, beads, and more complex structures for orthopedic applications. Porous CS materials can be obtained simply by freezing and lyophilizing CS acetic acid solutions (Madihally and Matthew, 1999). During lyophilisation, drying removes ice crystals creating a porous structure, where pore size can be regulated by a tight control of the temperature. Electrospinning can create CS nanofibers that thanks to their unique characteristics (including high surface area to volume ratio, porosity, permeability, stability, and similarity to that of ECM) have a significant superiority over other morphologies (Balagangadharan et al., 2017).

##### Biocomposites for BTE

CS has also been applied as injectable biomaterial for BTE, for instance as composite formulation with β-TCP and platelet rich plasma (PRP) (Bi et al., 2010), which contains a number of growth factors (i.e., bFGF, PGDF, TGF-β, IGF, and VEGF). Besides inducing higher ALP activity and osteogenic markers expression *in vitro*, CS-TCP-PRP efficiently repaired osseous defects in goat tibiae with radiologically evident effects after 16 weeks. As for other natural polymers, compositions with reinforcements and stimulating factors (like HA cement, alginate, hyaluronic acid, calcium phosphate, PMMA, PLA, and growth factors) has been a promising strategy to overcome the mechanical weakness and the lack of osteoinductive properties (Liu et al., 2006; Thein-Han and Misra, 2009; Venkatesan and Kim, 2010; Saravanan et al., 2011). In particular, the association with HA and other bioactive ceramics has become very popular (Venkatesan and Kim, 2010). Mouse osteoblasts (MC3T3-E1) and L929 cells adhere well and proliferate in CS-calcium phosphate specimens (Xu and Simon, 2005; Oliveira et al., 2006). In scaffolds composed of CS and poly(butylene succinate) (PBS) with elevated porosity and interconnectivity (59 and 60.9%, respectively), and with pore diameter of 145 μm, the MSC viability and ALP activity increased during 3 weeks of *in vitro* culture, and elevated rate of bone formation was observed in murine cranial, critical-size bone defects (Costa-Pinto et al., 2012). Higher porosity (around 97%) with an average pore size of 100 μM was achieved in CS-alginate systems (Florczyk et al., 2013), where the addition of proteins and other factors, like bone marrow aspirate or BMP-2, demonstrated good bone regeneration in a calvarial defect rat model. In general, morphogenic factors and drugs can be delivered in a controlled fashion from CS by virtue of its favorable gelling and degradation properties. CS allows for slow release of BMP-2 and TGF-β-2 in coatings covering the allografts in experimental cranial critical size defect models (Canter et al., 2010). In another study, scaffolds fabricated from CS and bovine-derived xenograft (BDX) at 40:60 ratio supported the *in vitro* proliferation and differentiation of bone marrow-derived MSCs (BM-MSCs) from the human jaw (Zang et al., 2017), that can be intraoperatively collected from alveolar bone during dental surgery (i.e., crown lengthening surgery and wisdom tooth extraction). To treat not self-repairing bone defects (like burr holes in craniotomy), certain CS-based systems have been designed (Shirosaki, 2012). CS-γ-glycidoxypropyltrimethoxysilane (GPTMS) hybrid can be prepared via sol-gel method, and further completed with HA by soaking in an alkaline phosphate solution, promoting skull bone formation *in vivo* (Shirosaki et al., 2018). Long-term studies conducted over 2 to 3 years to assess the outcome of implantation of CS-siloxane hybrids in skull bone regeneration revealed that new regenerated tissues completely closed the defect, but with a thickness inferior to the normal skull thickness (Shirosaki et al., 2018). CS functioned as effective system for slow, controlled release of BMP-2 and TGF-β-2 in coatings covering allografts in experimental cranial critical size defect models (Canter et al., 2010).

##### Chemical modification

The chemical modification of CS is reported to stimulate cell proliferation and ALP activity in different systems. For instance, CS glutamate and HA containing cultured osteoblasts from bone marrow aspirate efficiently repaired bone defects in 8 mm diameter cranial defects in rat calvaria (Mukherjee et al., 2003), as assessed in terms of mineral density found in the lesion sites. In another study, the immobilization of peptides was shown to contribute to bone formation (Qu et al., 2010): when decorated with RGD peptides, CS-HA scaffolds displayed 88.4% porosity with average pore size of 400 μm, and promoted osteogenesis via enhanced cell adhesion. In fact, the observation of significantly higher ALP activity suggests that engineering of optimized cell attachment sites is highly beneficial to the differentiation.

##### Biocomposites with nano-objects

CNTs have emerged as a special class of reinforcement fillers for CS nano-composites, thanks to their ability to drastically increase the mechanical strength (Wang et al., 2005). Moreover, they were found to have good bone tissue compatibility and capability to accelerate bone formation under stimulation with recombinant hBMP-2 (Usui et al., 2008). The incorporation of metallic nano-objects has also been reported. Doping with inert TiO_2_ nanoparticles transforms CS into highly porous, brittle and effective BTE substitute with a density (1.2870 g/cm^3^) comparable to that of dry bone (0.8–1.2 g/cm^3^) (Kumar, 2018).

##### Matrices for gene delivery

Much of the appeal of CS relies on the presence of repeating units rich in primary amine groups easily becoming protonated under acidic conditions. Thanks to this feature, CS became a relevant vector for non-viral gene delivery transfecting a number of cell types (Raftery et al., 2013). In fact, the cationic nature of CS elicits an efficient complexation with DNA molecules making it an ideal candidate for gene delivery (Di Martino et al., 2005). Successful CS-pDNA complexation and sustained transfection of MSCs makes CS a promising vector also for gene activated matrices (GAMs), which are scaffolds engineered to provide a direct and sustained delivery of nucleic acids ensuring efficient and durable cell transfection *in situ* (Raisin et al., 2016; D'Mello et al., 2017; Lin et al., 2017). In particular, the encapsulation of plasmids into nano or micro-particulate CS-systems to be loaded within scaffolds could offer significant spatiotemporal control on the activity of the encoded biofactors (Peng et al., 2009).

##### Current use

By virtue of its water retention, protein adsorption, mechanical strength, porosity, biocompatibility, biomineralization, and biodegradability, CS has drawn attention in BTE. However, its actual applicability is limited by some disadvantages, including immunogenicity and arduous electrospinning processing (Bellich et al., 2016). Moreover, further studies focusing on long-term *in vivo* application are still needed to validate the *in vitro* findings regarding CS properties.

#### Alginate

##### Structure and characteristics

As the most abundant marine biopolymers, alginates comprise a broad family of polysaccharides found in brown seaweeds. Even if they can be produced also by some bacteria (e.g., Azotobacter and Pseudomonas species), the cell wall and intracellular space of seaweeds (Laminaria sp., Macrocystis sp., Lessonia sp., and others) remain the major sources. Alginates are linear unbranched polymers containing β-(1 → 4)-linked D-mannuronic acid (M) and α-(1 → 4)-linked L-guluronic acid (G) residues. The overall polysaccharide sequence contains blocks of consecutive G and M residues, or alternating MG residues. Sodium alginate is the main form of alginate used. The ability of alginates to form soft hydrogels in the presence of Ca^2+^ relies on the participation of G-blocks in intermolecular cross-linking with divalent cations (Venkatesan et al., 2014b).

##### Manufacturing and properties of alginate scaffolds for BTE

Alginates can be shaped into a number of soft biomaterials including films, nanoparticles, foams, elastic gels, fibers, and multilayers stable in physiological conditions, which ensures the preservation of cell viability and function. Sodium alginate hydrogel was used as the carrier for dual delivery of BMP-2 and bFGF showing a sustained release ability supporting the proliferation and osteogenic differentiation of BM-MSCs inside nano-composite polymeric scaffolds (PLGA-PCL-nanoHA) loaded with vascular stents for large bone defect regeneration in rabbit mandibles (Su et al., 2013). Other alginate composites have been investigated, complemented with polymers (PLGA, PEG, and chitosan), proteins (collagen and gelatin), ceramic, biosilica, bioglass, and peptides (Venkatesan et al., 2015). Apart from physico-mechanical improvement, composites impart more marked biological effects on the seeded cells, in terms of cell affinity, osteogenic differentiation, and biomineralization. Scaffolds with highly porous and interconnected structures were manufactured by subjecting alginate-HA composite to internal gelation followed by freeze-drying (Marsich et al., 2013). In 2010, Suárez-González et al. described a simple method to control the nucleation of a bone-like HA mineral onto macroporous alginate scaffolds by incubation in modified SBFs for 4 weeks (Suárez-González et al., 2010), and reported improved MSC attachment. Freeze drying is the dominant technique to manufacture CS-alginate hybrids for BTE (Venkatesan et al., 2014b), whereas *in situ* co-precipitation elicits the integration of HA crystals (Jin et al., 2012). Rapid bone formation and vascularization were observed in these hybrids, characterized by enhanced mechanical strength and structural stability (Li et al., 2005). The addition of MSCs and BMP-2 is expected to generate injectable materials able to induce new bone formation in a clinical context (Park et al., 2005). Recently, a polypyrrole-alginate blend was incorporated into chitosan via lyophilisation to obtain a scaffold supporting the growth of MG-63 cells under electrical stimulation within a bioreactor system, to evaluate the role of a substrate endowed with conducting properties in bone regeneration (Sajesh et al., 2013).

##### Biocomposites with nano-objects

Among antimicrobial agents, silver induces disruption of bacterial cell membranes and inhibits DNA replication, enzymatic activity, and ATP production (Rai et al., 2001; Lara et al., 2011). Due to the high surface-to-volume ratio, nano-objects display a reactivity higher than the bulk element. Therefore, shaping the silver into nanoparticles further augments its bactericidal activity on different strains of clinical relevance (Rai et al., 2001; Alt et al., 2004; Chaloupka et al., 2010). Silver nanoparticles adsorbed on top of alginate scaffolds exert a strong bactericidal effect against both Gram+ and Gram- bacterial strains (Marsich et al., 2013). With a total porosity of around 94% and pore size of 130–170 μm, tricomponent scaffolds consisting of alginate, HA, and CNTs demonstrated enhanced proliferation, differentiation, and attachment of an osteosarcoma cell line (MG-63), and were therefore suggested for BTE applications (Rajesh and Ravichandran, 2015). A nanocomposite scaffold of GO, gelatin and alginate with high swelling (~700%) and slow biodegradation rate (~30% in 28 days) was obtained, where MSCs displayed elevated expression of osteoblast transcription factors (Runx2 and OCN) and ALP activity, suggesting good osteoinductivity (Purohit et al., 2019).

##### Micro-constructs

By aggregation, it is possible to prepare highly porous scaffolds from alginate microbeads and microfibers, where the biological tissue development benefits from the open structure with positive outcome on vascularization, oxygenation, cell migration, adhesion, and proliferation (Valente et al., 2012). Indeed, alginate is well-suited for the production of microspheres for the encapsulation and delivery of cells and proteins (Somo et al., 2017; Dhamecha et al., 2019). Single layered and multi-layered microbeads can assemble from alginate at neutral pH and mild temperatures through conventional external gelation protocols, in which vortexing, homogenization, ultrasonication, spray drying, or other instrumental manipulation differentially affect the overall particle characteristics (Dhamecha et al., 2019). Alginate microspheres encapsulating adipose-derived stem cells (ADSCs) or human osteoprogenitors (HOP) from BM-MSCs co-cultured with human umbilical vein endothelial cells (HUVECs) were made osteogenic and angiogenic by enrichment with PRP or grafting with RGD peptides (Grellier et al., 2009; Man et al., 2012). These systems are ideal candidates for the development of micro-invasive bone regeneration applications. Qiao et al. seeded mouse osteoblasts into alginate-CS microcapsules, complexed with calcium phosphate cement (CPC) to assess the osteogenic potential of the resulting paste and trace the implanted cells *in vivo* (Qiao et al., 2013). After 4 weeks of subcutaneous implantation, new collagen formation, lamellar bone-like mineralization and angiogenesis were observed, while at 8 weeks collagen expansion and osteoid-like structures were noticed. Similarly, human embryonic stem cells-derived MSCs were encapsulated in alginate microbeads inside macroporous CPC constructs, holding promise for bone regeneration in a wide range of orthopedic and maxillofacial applications (Tang et al., 2012). Moshaverinia et al. conducted *in vitro* studies to assess viability and osteogenic differentiation of MSCs from periodontal ligament and gingiva (PDLSCs and GMSCs), encapsulated into oxidized alginate microbeads constituting an injectable and biodegradable scaffold for BTE (Moshaverinia et al., 2012). In an attempt to mimic the composition of bone, gradual mineralization was also achieved by co-immobilizing stem cells and ALP into alginate beads (Westhrin et al., 2015).

##### Modified alginate with advanced biological functions

Alginate has been structurally modified to host a variety of functionalities. For instance, peptides containing RGD or PHSRN (proline-histidine-serine-arginine-asparagine) sequences from fibronectin were grafted on alginates in order to create functionalized gels that more closely recapitulate the chemistry of natural cell adhesive proteins and may be useful in developing optimized BTE scaffolds (Nakaoka et al., 2013). In another study, alginate scaffolds seeded with human BM-MSCs demonstrated enhanced bone regenerative capability in critically-sized femur defects in mice through a fine temporal control on the release kinetics of bioactive factors (Kanczler et al., 2010a). In fact, alginate-PLA hybrids, generated through a supercritical CO_2_/alginate entrapment technique, were characterized by distinct biodegradation rates of the two components. As a consequence, they could release selected factors at different rates, with a rapid delivery of VEGF by alginate fibers as readily degradable carrier in contrast to a slower release of BMP-2 occurring from the synthetic polymer. Kolambkar et al. went a step further by designing a strategy for spatiotemporal control of growth factor delivery based on a hybrid system in which an injectable alginate hydrogel was used for rhBMP-2 delivery, while an electrospun nanofiber mesh served for guiding bone regeneration in critically-sized segmental defects in a rat model (Kolambkar et al., 2011).

##### Current use

Even though alginate has been declared safe for application in humans by the U.S. Food and Drug Administration (FDA) (de Vos et al., 2014; Xu and Lam, 2018), no devices for BTE have been commercialized so far. Interestingly, the use of alginates as bioinks for bioprinting is thought to be a valid opportunity for these hydrogels to expand their application in BTE (Hernández-González et al., 2020).

#### Silk Proteins

##### Structure and characteristics

Silk is a protein fiber secreted by arthropods like silkworms and spiders. It is composed of a structural core component, fibroin, and a hydrophilic protein coating made of sericin. The silk fibroin (SF) is a fibrous protein owing unique mechanical properties, environmental stability, morphologic flexibility, tunable proteolytic biodegradability, along with the ability to support the osteogenic differentiation of MSCs.

##### Manufacturing and properties of silk scaffolds for BTE

SF can be molded into diverse forms, chemically modified and combined synergistically with other minerals and polymers (Melke et al., 2016; Yao et al., 2016; Bhattacharjee et al., 2017). Films are obtained by layer-by-layer deposition or casting, whereas hydrogels by sol-gel transition or crosslinking. Fiber deposition and electrospinning are the most conventional options to shape SF into non-woven mats. Finally, particulate leaching, gas foaming, rapid prototyping (3D printing) and freeze drying render can generate 3D porous structures scaffolds (Bhattacharjee et al., 2017). Macroporous SF scaffolds, fabricated by water- or solvent-based procedures, displayed new bone formation at 8 weeks after implantation in defects of tibia and humerus cancellous bone in a sheep model (Uebersax et al., 2013). By adding BMP-2 into SF solution before electrospinning, a fibrous biomaterial with improved repair ability was created by Li et al. (2006).

##### Biocomposites, particles, and chemical modification

Since SF porous scaffolds and hydrogels often fail to match the mechanical demands of BTE, reinforcement strategies have been adopted in many works. Beyond conventional reinforcing materials (HA, BGNs, polymers, etc.), also SF microparticles were employed to such scope, increasing the compressive modulus of SF hydrogels over6-folds in a 1:2 (matrix:particle) mixture, which in turn enhanced the osteogenic performance of MSCs as well as the calcium adsorption (Rockwood et al., 2011). Moreover, silk particulate can function as template for mineralization and sustainable delivery of growth factors. SF modification with PRP, endothelial nitric oxide, IGF-I, or adenoviruses carrying BMP-7-encoding DNA has been proposed to prompt and strengthen silk scaffolds' osteointegration (Bhattacharjee et al., 2017). One limiting factor in the use of SF is the co-existence of sericin with fibroin in the native silk fiber, which may trigger antigenic reaction (Meinel and Kaplan, 2012). This shortcoming can be overcome by adjusting the silk processing procedure with physical and/or chemical modifications, or incorporation of other polymers/minerals (Bhattacharjee et al., 2017).

Also, the very same soluble sericin extracted from native silk fibers was considered in BTE for its ability to mediate the formation of HA crystals in SBF, in turn stimulating adhesion, proliferation, and osteogenic differentiation of human BM-MSCs (Yang et al., 2014; Jiayao et al., 2017).

##### Current use

Even though, due to continuing fast-paced progress, several silk-devices are expected to be added to practice in wound dressing or orthopedic implants, currently none has yet proceeded to human trials.

#### Hyaluronic Acid

##### Structure and characteristics

Hyaluronic acid (HAc) is a glycosaminoglycan containing repeating disaccharide units of N-acetyl-D-glucosamine and D-glucuronic acid. Synthesized by hyaluronan synthases, it is a primary component of the ECM of the human connective tissue and the simplest one among the various GAGs (Hemshekhar et al., 2016). Thanks to its biocompatibility, ease of chemical functionalization, degradability, hydrophilicity, non-immunogenicity, and presence in the cytoplasm of osteoprogenitors, HAc has been used in BTE (Zhao et al., 2016).

##### Chemical modification

The solubility and availability of reactive functional groups facilitate HAc chemical modifications. For instance, thiol-modified HAc combined with polyethylene glycol (PEG) provided biphasic release kinetics of BMP-2 accounting for an initial burst followed by a sustained release and leading to ectopic bone formation (Bhakta et al., 2013). In another research, by conjugating 2-aminoethyl methacrylate (AEMA) to HAc via amide bonds, followed by mixing with a cytocompatible photoinitiator (Irgacure D-2959) and irradiation under 365 nm UV light, Bae et al. prepared photo-cured hydrogels carrying the growth and differentiation factor 5 (GDF5) and simvastatin, which significantly improved osteogenesis (Bae et al., 2011, 2014).

##### Current use

It was found that Hyaloss^TM^, a HAc-based matrix, mixed with autologous bone accelerated bone formation and remodeling in post-extractive sites (Baldini et al., 2010), whereas ChronOS inject, a resorbable bone void filler made of HAc and TCP, proved to be successful in curing benign bone cysts (Joeris et al., 2010). Nevertheless, even if increasing attention is given on a clinical use of HAc, well-designed clinical randomized controlled trials with long follow-ups are still needed to judge its actual effectiveness in dentistry and bone repair (Zhao et al., 2016).

#### Other Natural Polymers

Other natural polymers widely used for TE of other organs are explored in bone repair.

##### Fibrin

Produced from fibrinogen, fibrin is a protein matrix containing sites for cell binding, which has been investigated as a substrate for cell adhesion, spreading, migration, and proliferation (Ahmed et al., 2008). Besides being immune-compatible and owing haemostatic, chemotactic, and mitogenic properties, fibrin enables the fabrication of completely autologous scaffolds, given that its precursors, thrombin and fibrinogen, can be extracted from the peripheral blood of patients (Noori et al., 2017). For instance, autologous fibrin glue has found extensive application in regenerative maxillofacial surgery (Khodakaram-Tafti et al., 2017).

##### Self-assembling peptides

Hydrogels self-assembled from gelation of self-complementary amphiphilic peptides are a novel class of TE biomaterials. Peptides can easily be modified to contain bioactive motifs, such as phosphoserine residues or RGDS peptides beneficial for mineralization and cell adhesion respectively (Mata et al., 2010; Visser et al., 2016). TCP minerals and peptide hydrogels act synergistically to enhance bone regeneration in a non-critical sized defect in rat femurs (Amosi et al., 2012). A commercially available peptidic hydrogel, Puramatrix™, can support the osteogenic differentiation route of dedifferentiated cells derived from subcutaneous fat through minimally invasive procedures. Varying the concentration of Puramatrix™ in the hydrogel enables to control the matrix stiffness, affecting MC3T3-E1 osteoprogenitor cell behavior (cell elongation and osteogenesis) (Kishimoto et al., 2011; Marí-Buyé et al., 2013).

##### Keratin

Keratin comprises a family of structural, filament-forming proteins found in epidermal and corneous tissues emerging as alternative for design of BTE scaffolds and strategies (Tachibana et al., 2005). The main sources of extraction are human hair, wool, and feathers (Rajabi et al., 2020). For example, keratin osteoconductive porous scaffolds can be engineered (Arslan et al., 2017). Carriers with tunable erosion rates were formulated by varying disulfide crosslinking ratios of oxidatively (keratose) to reductively (kerateine) extracted keratin. Such carriers released rhBMP-2 *in vitro* and mediated heterotopic bone formation in mice (Cohen et al., 2018).

##### Gellan gum

Gellan gum derived from bacterial fermentation (Sphingomonas group) forms injectable and thermoreversible hydrogels with tunable mechanical properties useful for preparation of bilayered scaffolds for osteochondral regeneration (Pereira et al., 2013; Manda et al., 2018).

##### Spongin

As analogous to vertebral collagen type XIII, spongin, the main organic component of sponge fibrous skeletons, has emerged as excellent alternative source of collagen proteins for BTE scaffolds due to the lower risk of transmission of infection-causing agents (Granito et al., 2016). Human osteoprogenitors can attach, aggregate, and grow at sustained rate on collagen fibers of the skeleton of an undetermined species of Spongia (Spongiidae family) (Green et al., 2003).

Finally, other polysaccharides (like dextran, cellulose, starch, carrageenans, or agarose) and proteins (like heparin or chondroitin sulfate) with relevance for BTE, are listed in Table 3 (Chung and Park, 2007; Martins et al., 2009; Garg et al., 2012; Khan and Ahmad, 2013; Sayin et al., 2014; Shi et al., 2016; Farrugia et al., 2018; Nikpour et al., 2018; Sofi et al., 2018; Witzler et al., 2019; Yegappan et al., 2019). Physico-mechanical characteristics of various composites based on natural polymers are compared in Table 4.

## Future Perspective and Conclusions

Being very similar or identical to endogenous macromolecules, natural polymers are optimal constituents for implantation, not only because the biological environment is prepared to recognize and metabolically process them, but also because they enable to tailor the biomaterial functions at the molecular level (Yannas, 2004; Jahan and Tabrizian, 2016). The fact that natural polymers are degraded by naturally occurring enzymes virtually guarantees that after implantation, the constructs will be eventually resorbed through physiological processes (Yannas, 2004; Bhatia, 2016). Cross-linking and other strategies of chemical modification enable the designer to control the lifetime of the implant, with important impact in the applications where scaffolds are supposed to deliver a specific function over a precise period of time before being replaced by newly formed tissue (Lee and Shin, 2007; Lee and Yuk, 2007; Bhatia, 2016; Salehi-Nik et al., 2017). However, this natural feature carries certain limitations. As compared to the synthetic polymers, the natural ones owe much more complex structures requiring elaborated technological manipulation, and almost inevitably, their modification, as well as the physico-chemical methods to isolate them from tissues, can significantly alter their native conformation (Yannas, 2004). Accounting for an enormous variability among species and functional compartments (i.e., species and tissue specificity), macromolecules of animal derivation also make it necessary to implement stringent control procedures on the nature of the raw material in order to ensure adherence to quality and uniformity specifications among different batches. Due to their similarity to natural substances, TE proteins are strongly immunogenic, such that modification of antigenic determinants is often needed to impart integrity and long duration of the implant (Yannas, 2004; Titorencu et al., 2017). Besides being far more immunogenic than sugars and other polymers, proteins also pose a precise challenge in terms of manufacturing. The pyrolytic modification and the decomposition usually occurring when exceeding the melting temperature preclude the application of thermoplastic processing at high temperature (like melt extrusion), and make it necessary to implement alternative methods for extrusion at room temperature (Yannas, 2004; Wang et al., 2020). On balance, these opposite characteristics result in qualifying the natural polymers as intriguing materials for bioengineering and transplantation, providing unprecedented solutions in BTE (Oliveira et al., 2018).

Based on the collection of studies herein reviewed, one can distinguish the following promising research directions in natural polymeric BTE constructs: (i) strategies of controlled delivery of bioactive molecules to accelerate bone healing, (ii) modification of polymers to optimize the cell-matrix interactions and shape the scaffold topography, and (iii) engineering of GAMs to integrate advanced principles of gene therapy in the science of scaffold design.

One of the most relevant perspectives in polymer science is represented by the creation of functional coatings improving the properties of biomaterials, such as bioceramics, which are ideal bone regeneration platforms by virtue of their drug-delivery ability and apatite-like formation (Mohamad Yunos et al., 2008; Baino et al., 2015). As the surface instability and inherent brittleness in bioglass foams compromise their mechanical strength and cytocompatibility, polymer coatings can be introduced to reduce the degradation rate or improve the interaction with the biological surrounding (Peroglio et al., 2007; Mohamad Yunos et al., 2008; Rehorek et al., 2013). Even though synthetic polymers held a predominant role thus far, the natural ones have started to conquer space in this territory (Mohamad Yunos et al., 2008; Rehorek et al., 2013; Iviglia et al., 2016; Wang J. et al., 2016). For instance, a biodegradable layer of PHB produced by bacteria isolation considerably increased the bioactivity and doubled the compressive strength of glass-ceramics without altering the interconnectivity of their pore structure (Bretcanu et al., 2009). Coating with pectin-chitosan polyelectrolytes elicited the controlled release of antibiotics from porous scaffolds in order to contrast periprosthetic infections (Iviglia et al., 2016), whereas blending silk with mesoporous bioglass scaffolds was beneficial to stromal cell attachment, proliferation and osteogenic differentiation (Wu et al., 2010). Besides displaying values of Young's modulus and fracture toughness similar to those of native bone, bioceramics vacuum-coated with alginate promoted osteoblast adhesion and maturation (Torres et al., 2013). The laminin-coating, in addition to amine-surface modification, promoted cell colonization of foams (Tan et al., 2003). Immobilization of natural polymers onto synthetic coating is also possible and functional to enhance the scaffold properties (Wang X. et al., 2016). Major advances in this field of polymer-based BTE are expected in a near future.

In addition, it has been well-documented that the incorporation of nano-sized inorganic particles in natural polymer composites not only leads to incremental cellular adhesion, but can also improve the polymer mechanical properties and osteoconductive ability to a higher extent than micro-sized fillers. In fact, osteogenic cells optimally interact with nanophase minerals and proteins, since they provide larger surface area and create nanoscale roughness (Dobbenga et al., 2016).

Another eminent aspect in the field of BTE scaffold design concerns the opportunity to use the constructs to harness SC fate toward specific lineages. Accordingly, cues for osteogenic induction can be integrated in the scaffold structure and delivered to cells via physical stimuli. For instance, the mechano-transduction signaling pathways in SCs can be activated by tagging magnetic nanoparticles to mechanosensitive membrane receptors and actuating these effectors through electromagnetic fields, inducing osteogenesis (Kanczler et al., 2010b).

Future investigations should also aim at preparing biomaterials with degradation rate matching the bone regeneration rate, integrated with strategies for stimulating scaffold vascularization (e.g., co-cultures, growth factors, etc.) and bioreactor technologies to streamline and improve pre-implantation culture. Also, novel procedures for cell biophysical stimulation should be implemented to ease access to clinical use. In fact, despite several successful *in vitro* and *in vivo* trials of constructs based on natural polymers, few products are now commercially available or under clinical investigation, such that the translational benefits remain distant from potential patients. Currently, the majority of biological scaffolds with actual relevance in clinical use are those comprising collagens, such as absorbable collagen sponges, porous scaffolds, HA composites and gel foams (Zeng et al., 2018). It has to be mentioned that cultural and religious traditions may limit the acceptance of collagens and other polymers of animal origin: for instance, the Muslim and Jewish beliefs forbid the consumption of pig meat, identifying swine-derived biomaterials as banned. Similarly, followers of the Hindu faith may refuse bovine surgical products. In modern multicultural societies, the implantation of animal polymers must be anticipated by the informed consent process to avoid religious distress and possible litigation. Nevertheless, the rhetorical-cultural challenge in getting patients overcoming the prejudices about the material's uncleanliness, impurity, and offensiveness to the religious sensibility cannot be ignored even when developing the scaffolds to the clinical grade in order to predict their actual applicative impact.

In conclusion, it is clear that a deeper knowledge about the dynamics of the bone tissue microenvironment should allow for a proper simulation, likely meeting the open challenges still limiting the natural polymers from entering into BTE clinically. However, these polymers offer a unique chance to create biomimetic biocompatible microenvironments for cells of the osteogenic niche. The strategies for engineering their properties are expected to further augment their clinical potential and become determinant in the design of efficient regenerative matrices to repair the skeletal lesions in the future.

## Author Contributions

MF conceived the original idea of the manuscript, structured the outline, wrote the main text and prepared figures and tables. GB, MC, and AS edited the final manuscript. All authors approved it for publication.

## Conflict of Interest

The authors declare that the research was conducted in the absence of any commercial or financial relationships that could be construed as a potential conflict of interest.
